# Contribution of Epigenetic Modifications to the Decline in Transgene Expression from Plasmid DNA in Mouse Liver

**DOI:** 10.3390/pharmaceutics7030199

**Published:** 2015-08-07

**Authors:** Lei Zang, Makiya Nishikawa, Mitsuru Ando, Yuki Takahashi, Yoshinobu Takakura

**Affiliations:** Department of Biopharmaceutics and Drug Metabolism, Graduate School of Pharmaceutical Sciences, Kyoto University, Sakyo-ku, Kyoto 606-8501, Japan; E-Mails: zanglei80@yahoo.co.jp (L.Z.); andou.mitsuru.8s@kyoto-u.ac.jp (M.A.); ytakahashi@pharm.kyoto-u.ac.jp (Y.Takah); takakura@pharm.kyoto-u.ac.jp (Y.Takak)

**Keywords:** transgene expression, CpG, DNA methylation, histone deacetylation, hydrodynamic injection

## Abstract

Short-term expression of transgenes is one of the problems frequently associated with non-viral *in vivo* gene transfer. To obtain experimental evidence for the design of sustainable transgene expression systems, the contribution of epigenetic modifications to the decline in transgene expression needs to be investigated. Bisulfite sequencing and reactivation by hydrodynamic injection of isotonic solution were employed to investigate methylation statues of CpG in transiently expressing plasmid, pCMV-Luc, in mouse liver after hydrodynamic delivery. The cytosines of CpGs in the promoter region of pCMV-Luc were methylated in mouse liver, but the methylation was much later than the decline in the expression. The expression from pre-methylated pCMV-Luc was insensitive to reactivation. Neither an inhibitor of DNA methylation nor an inhibitor of histone deacetylation had significant effects on transgene expression after hydrodynamic injection of pCMV-Luc. Partial hepatectomy, which reduces the transgene expression from the non-integrated vector into the genome, significantly reduced the transgene expression of human interferon γ from a long-term expressing plasmid pCpG-Huγ, suggesting that the CpG-reduced plasmid was not significantly integrated into the genomic DNA. These results indicate that the CpG-reduced plasmids achieve prolonged transgene expression without integration into the host genome, although the methylation status of CpG sequences in plasmids will not be associated with the prolonged expression.

## 1. Introduction

Therapeutic effects of *in vivo* gene therapy depend on the spacio-temporal distribution of the transgene products. The most important parameter associated with the *in vivo* gene transfer of secretory proteins is the temporal profile of the transgene expression or, in other words, the level and duration of the expression [1,2], because the number of transfected cells would be of less importance for secretory proteins compared with intracellular proteins. The level and duration of the expression are the results of complicated intracellular events, including transcription and translation, and the mechanisms underlying them have not fully been explored yet. The development of hydrodynamic delivery of naked plasmid DNA has almost solved the problem of low level of transgene expression as demonstrated in a comparison with adenoviral vectors [3]. Unfortunately, hydrodynamic injections are frequently associated with transient transgene expression, especially when using plasmid vectors containing cytomegalovirus (CMV) promoter, one of the strongest promoters used to date.

Short-term expression can be due to the degradation or clearance of plasmid DNA. Because plasmid DNA consists of natural phosphodiester deoxypolynucleotides, it is easily degraded by DNases. Kawabata *et al.* reported that naked plasmid DNA degrades in mouse blood with a half-life of less than 10 min [4]. However, plasmid DNA could exist for a long period of time once it has entered into the nucleus. It has been reported that plasmid DNA is present in the nucleus of mouse liver for at least 28 days after *in vivo* gene transfer even when the transgene expression from the plasmid DNA has almost ceased [5,6]. In addition, silenced transgene expression can be reactivated by several stimuli, including viral infection, ischemia reperfusion, and hydrodynamic injections, even 6 months after the original *in vivo* gene transfer [2,7,8,9]. Integration of plasmid DNA into the genome would also result in a sustained transgene expression [10]. These experimental results indicate that the degradation of plasmid DNA is not a major mechanism at least for the rapid decline in transgene expression observed within several days of gene transfer.

The presence of silenced or inactive plasmid DNA suggests some mechanisms that inhibit transgene expression from plasmids in the nucleus. One of the most frequently discussed mechanisms is the methylation of deoxycytidyl-deoxyguanosine (CpG) dinucleotides in plasmid DNA [11]. Yew *et al.* developed the GZB plasmid vector in which a synthetic CMV promoter/enhancer with no CpG sequences was used to drive cDNA [12]. In addition, we reported that a plasmid expressing murine interferon (IFN)-γ under the control of CpG-free human elongation factor (EF)-1 promoter produced a sustained level of IFN-γ compared with that with a CMV promoter after hydrodynamic injection into mice [13,14,15,16,17,18]. Another suggested mechanism includes the interaction of histones with plasmid DNA. Genomic DNA binds to histones or other nuclear proteins in the nucleus to form the nucleosome [19]. Deacetylation of histone has also been suggested to be involved in the decline of transgene expression from plasmid vectors [20]. Finally, if plasmid vectors were inserted into the genome, the expression might be controlled differently from the expression from episomal plasmid vectors.

To answer these questions, we have examined whether epigenetic modifications take place in plasmid vectors that have been delivered to mouse liver. The methylation status of the plasmids has been evaluated, and the involvement of histone deacetylation and the integration into the genome have been examined in mice.

## 2. Experimental Section

### 2.1. Mice

Four-week-old male ICR mice, approximately 20 g in body weight, were purchased from Japan SLC, Inc. (Hamamatsu, Japan), and were maintained under conventional housing conditions. The protocol for the animal experiments was approved by the Animal Experimentation Committee of Graduate School of Pharmaceutical Sciences, Kyoto University (the protocol number, #2010-17; approval date, 10 March 2010).

### 2.2. Plasmid DNA

pcDNA3.1 and pcDNA3 were purchased from Invitrogen (Carlsbad, CA, USA) and pCpG-mcs was obtained from InvivoGen (San Diego, CA, USA). pCMV-Luc and pCpG-Luc were constructed as previously reported [13,21]. pCpG-Huγ was constructed by inserting the *Bgl*II/*Nhe*I human IFN-γ cDNA fragment amplified by PCR into the *Bgl*II/*Nhe*I site of pCpG-mcs [22]. pCMV-Huγ was constructed by inserting the human IFN-γ fragment from pCpG-Huγ digested by *Stu*I/*Nhe*I into the *Eco*RV/*Xba*I site of pcDNA3. The plasmids used in this study are summarized in Table 1.

**Table 1:** Plasmid DNA used in this study.

Plasmid	Promoter	Backbone	cDNA
pCMV-Luc	CMV	pcDNA3.1	Luciferase
pCpG-Luc	EF1	pCpG-mcs	Luciferase
pCMV-Huγ	CMV	pcDNA3	Human IFN-γ
pCpG-Huγ	EF1	pCpG-mcs	Human IFN-γ

### 2.3. In Vivo Gene Transfer and Reactivation of Transgene Expression

For gene transfer to the liver, mice received a tail vein injection of plasmid DNA dissolved in a large volume of saline (8% volume of body weight) over 5 s [23]. In some cases, mice received another large volume injection of saline (8% volume of body weight) over 5 s to reactivate the silenced expression in mouse liver [2].

### 2.4. Luciferase Assay

At indicated periods after injection, mice were killed, and the livers were harvested and homogenized in 5 mL/g of a lysis buffer (0.1 M Tris, 0.05% TritonX-100, 2 mM EDTA, pH 7.8). The homogenates were centrifuged at 10,000× *g* for 10 min at 4 °C. Then, 5 μL of each supernatant was mixed with 50 μL luciferase assay buffer (Picagene, Tokyo Ink, Tokyo, Japan) and the chemiluminescence was measured with a luminometer (Lumat LB 9507, EG&G Berthold, Bad Wildbad, Germany).

### 2.5. Extraction of DNA

DNA was extracted from liver using a DNeasy Blood and Tissue Kit (QIAGEN, Hilden, Germany) according to the manufacturer’s instructions.

### 2.6. Methylation of Cytosine Residues in Plasmid DNA

Plasmids (pCMV-Luc, pCpG-Luc) were treated with *Sss*I CpG methylase (New England Biolabs, Ipswich, MA, USA), which methylates cytosine residues of CpG dinucleotides. Mock-methylated plasmids, which were treated in the same manner as methylated ones without addition of the methylase, were used as controls.

### 2.7. Bisulfite Sequencing

Methylation of plasmid DNA was determined by bisulfite sequencing [24]. Bisulfite modification was performed on samples containing 1 μg DNA (including genomic DNA and plasmid DNA) extracted from mouse liver using BisulFast^®^ DNA modification kit (TOYOBO, Osaka, Japan) according to the manufacturer’s instructions. PCR primers were designed by changing all non-CpG cytosine residues in the sequence to thymine residues in order to amplify the bisulfite-treated DNA. Primers were selected from regions with no CpG nucleotides to amplify the methylated and unmethylated DNA equally. The following primers gave efficient and specific amplification of a part of the CMV promoter of pCMV-Luc containing 20CpGs: forward, 5′-GTTAATAGGGATTTTTTATTGA-3′; reverse, 5′-AAACTCTACTTATATAAACCTCCCACC-3′. Similarly, the following primers were used for the amplification of a part of the luciferase cDNA of pCMV-Luc containing 20 CpGs: forward, 5′-TGTTGGTGTTAATTTTATTTTTTTT-3′; reverse, 5′-CCAAAATATAACCATCCATCCTTAT-3′. Bisulfite-treated DNA was PCR-amplified using TaKaRa Ex Taq TM (Takara Bio Inc., Otsu, Japan). Cycling conditions were 5 min at 94 °C, then 40 cycles of 94 °C 30 s, 54 °C 30 s, 72 °C 30 s for the promoter part; and 5 min at 94 °C, then 40 cycles of 94 °C 30 s, 55 °C 30 s, 72 °C 30 s for the cDNA part. The PCR products were fractionated on 3% agarose gel and PCR products of the expected size were excised from the gel, purified and then cloned into pCR2.1 TOPO vector (Invitrogen, Carlsbad, CA, USA) according to the manufacturer’s protocol. Between 6–8 colonies that contained the insert were selected at random for each bisulfite reaction performed and sequenced to obtain a representative sampling DNA methylation pattern. The sequences of the amplified promoter and cDNA parts of pCMV-Luc were analyzed using a QUMA analysis tool (http://quma.cdb.riken.jp).

### 2.8. 5-aza-2’-Deoxycytidine and Trichostatin A Treatment

5-aza-2’-Deoxycytidine (5-AZA) (Sigma–Aldrich, St. Louis, MO, USA) was intraperitoneally injected into mice at a dose of 0.25 mg/kg body weight at 0, 2 and 4 days after hydrodynamic injection of pCMV-Huγ [25]. Trichostatin A (TSA) (Sigma–Aldrich) was dissolved in 50% DMSO to give a concentration of 1 mg/mL. Mice were given an intraperitoneal injection of TSA at a dose of 10 mg/kg body weight at 3 days after hydrodynamic injection of pCMV-Huγ. Control mice received an intraperitoneal injection of the same volume of the vehicle (50% DMSO).

### 2.9. Partial Hepatectomy

One week after hydrodynamic injection of pCpG-Huγ, mice were anesthetized with Nembutal (Dainihon Seiyaku, Osaka, Japan) and a midline incision was made on the abdomen. The left posterior lobes were excised to achieve a 37% hepatectomy, and the incision was closed with clips.

### 2.10. Measurement of Serum Concentration of Human IFN-γ

At indicated periods after gene transfer, 50 to 200 µL blood was collected from the tail vein. The blood samples were incubated at 4 °C for 2 h to allow clotting and then centrifuged at 8000× *g* to obtain serum. The concentration of human IFN-γ in the serum was analyzed by ELISA using commercial kits (Human IFN-γ Ready-SET-Go! ELISA kit, eBioscience, San Diego, CA, USA).

### 2.11. Statistical Analysis

Differences were statistically evaluated by Student’s *t*-test. The level of statistical significance was set at *p* < 0.05.

## 3. Results

### 3.1. Methylation Status of CMV Promoter and Luciferase cDNA of pCMV-Luc in Mouse Liver

**Figure 1:**
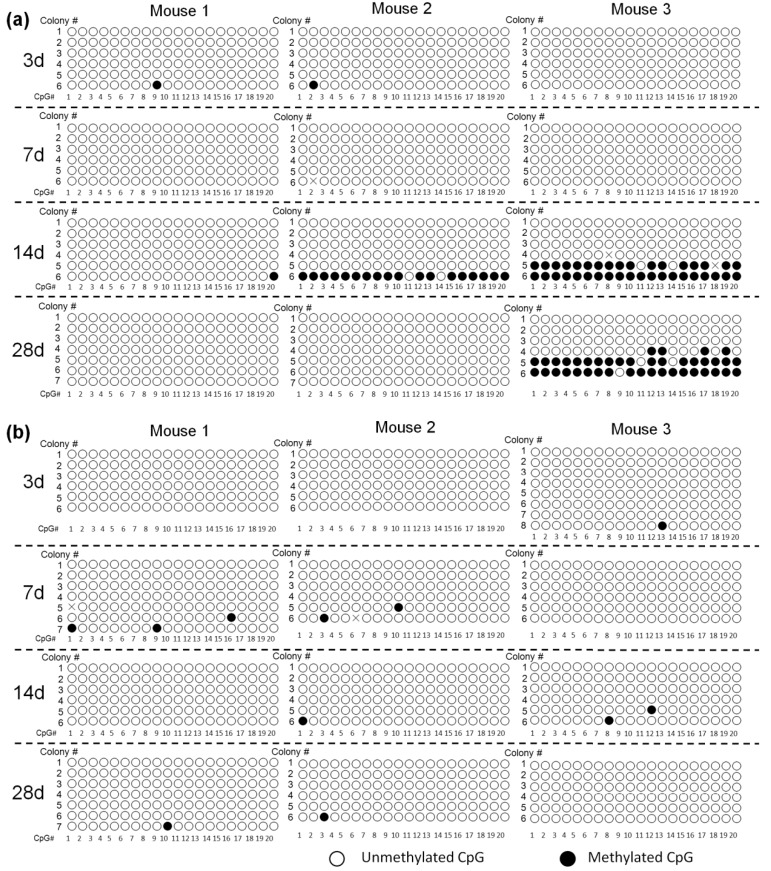
Methylation status of cytomegalovirus (CMV) promoter (**a**) and luciferase cDNA (**b**) in pCMV-Luc. Mice were hydrodynamically injected with 100 μg pCMV-Luc. DNA was extracted from mouse liver on day 3, 7 14 and 28 after the hydrodynamic injection of pCMV-Luc and subjected to bisulfite sequencing. Each circle represents one CpG dinucleotide. Six to eight colonies were randomly selected from the liver samples of each mouse, and were numbered. CpG # represents the location of CpGs in the amplified promoter or cDNA parts, with #1 being the nearest to the 5′-end. The positions that could not be read were indicated with X.

It was observed that, on average, about 95% of cytosines were methylated in the *Sss*I-treated plasmid DNA, whereas no cytosines were found to be methylated in the untreated one (data not shown), supporting the validity of the bisulfite sequencing method to estimate the methylation of plasmids. Figure 1a shows the methylation status of cytosine residues in the promoter region of pCMV-Luc. Few CpGs had been methylated 7 days after injection. On day 14, some of the CpGs in the region were detected to be methylated in samples isolated from two out of three mice. Several of the 6 colonies in the two mice showed methylation with a high frequency of 90%, 90% and 100%, respectively.

Then, the methylation status in the luciferase cDNA region was examined using the same DNA samples used in the above experiments (Figure 1b). The CpGs in this region were hardly methylated at least for the first 28 days after gene transfer.

### 3.2. Correlation of Methylation and Reactivation of Silenced Transgene Expression

**Figure 2:**
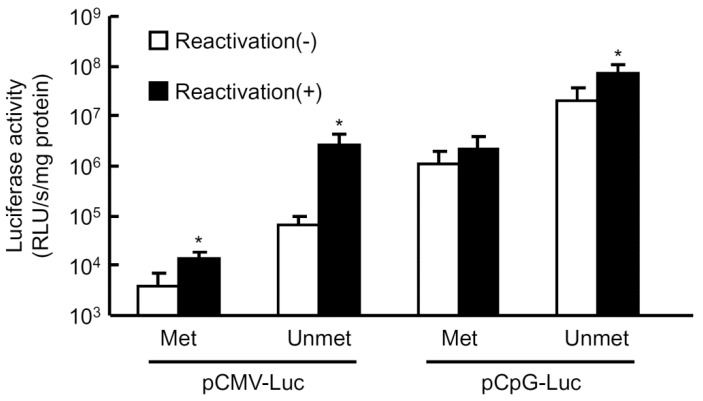
Reactivation of transgene expression in mouse liver by large-volume injection of saline. Methylated or mock-methylated plasmid DNA was hydrodynamically injected into mice at a dose of 0.3 pmol/mouse. Three days later, groups of mice received a large-volume injection of saline (8% *v*/*w* of body weight) to reactivate the transgene expression in the liver, and the remaining groups were left untreated. The luciferase activity in the liver was evaluated 12 h after the saline injection. The results are expressed as the mean ± SD of four mice. * *p* < 0.05 compared with the untreated group.

It has been reported that silenced transgene expression is reactivated by hydrodynamic injections of isotonic solutions [1,2]. However, it is not clear whether the reactivation is mediated by methylated or unmethylated plasmid DNA. To further clarify the correlation of methylation and silenced transgene expression, the reactivation experiment was carried out using both methylated and unmethylated plasmids. The pCMV-Luc or pCpG-Luc in either unmethylated or methylated form was injected into mice by the hydrodynamic injection method, and a large volume of saline was injected into the mice 3 days later to reactivate the silenced transgene expression (Figure 2). The level of expression from the methylated pCMV-Luc was much lower than that of the unmethylated counterpart, and the saline injection resulted in a moderate (about 3.7-fold) increase in the expression. On the other hand, the same treatment increased the expression up to about 40-fold when the unmethylated pCMV-Luc was administered. The increase in the expression produced by the second saline injection was 3.5- and 1.9-fold for the unmethylated and methylated pCpG-Luc, respectively. The difference in the luciferase activity between unmethylated and methylated pCpG-Luc would be due to the methylation of CpGs in the luciferase cDNA.

### 3.3. Effect of 5-AZA on the Transgene Expression of pCMV-Huγ

**Figure 3:**
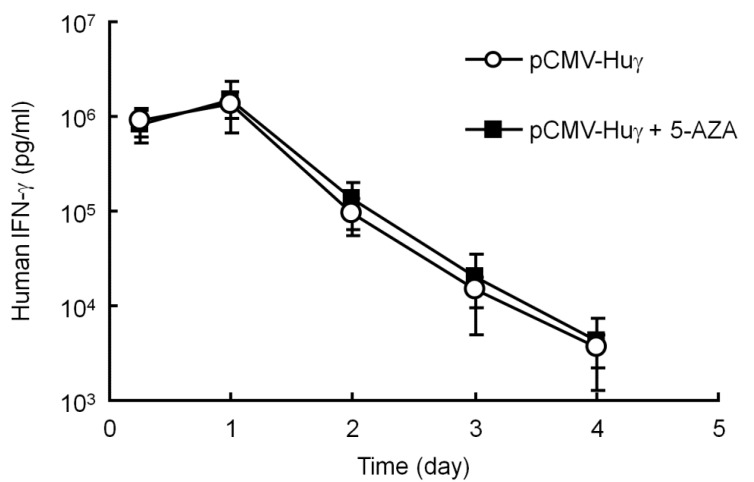
Effect of 5-aza-2’-deoxycytidine (5-AZA) on the serum concentration of human interferon (IFN)-γ after injection of pCMV-Huγ. pCMV-Huγ was hydrodynamically injected into mice at a dose of 20 μg/mouse. A group of mice received intraperitoneal injections of 5-AZA at a dose of 0.25 mg/kg every 2 days. The concentrations of human IFN-γ in the serum were measured at the indicated time points. The results are expressed as the mean ± SD of four mice.

The compound, 5-AZA, an inhibitor of DNA methylation, was employed to confirm whether plasmid DNA is methylated or not in mouse liver after hydrodynamic injection. pCMV-Huγ (a human IFN-γ expressing plasmid) was injected into mice by the hydrodynamic injection method with or without intraperitoneal injections of 5-AZA at a dose of 0.25 mg/kg every 2 days. Figure 3 shows the concentration of human IFN-γ in the mouse serum after gene transfer. There were no significant differences in the levels of human IFN-γ between the two groups, suggesting that pCMV-Huγ is hardly methylated during this period. Taken together with the results shown in Figure 1 and Figure 2, it is reasonable to conclude that plasmid DNA is hardly methylated in mouse liver after hydrodynamic injections at least for the first couple of weeks.

### 3.4. Effect of TSA on the Transgene Expression of pCMV-Huγ

**Figure 4:**
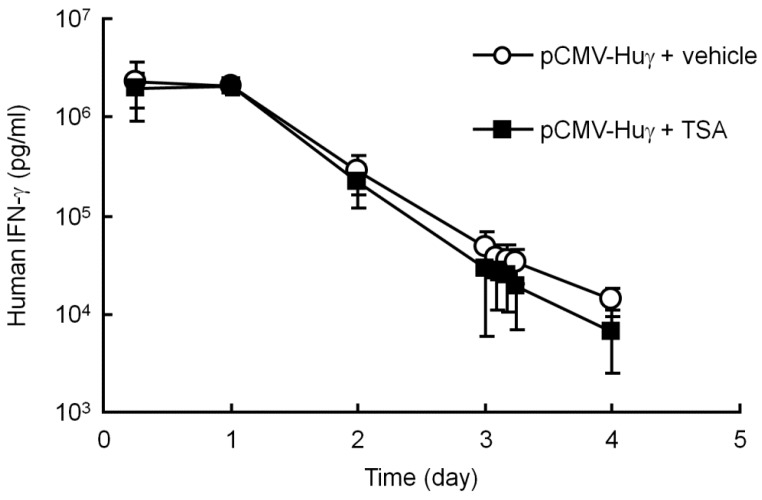
Effect of trichostatin A (TSA) on the serum concentration of human IFN-γ after injection of pCMV-Huγ. pCMV-Huγ was hydrodynamically injected into mice at a dose of 20 μg/mouse. On day 3, mice received an intraperitoneal injection of vehicle or TSA at a dose of 10 mg/kg. The concentrations of human IFN-γ in the serum were measured at the indicated time points. The results are expressed as the mean ± SD of four mice.

To test the possibility of the involvement of histone deacetylation in silenced transgene expression, TSA, a histone deacetylase (HDAC) inhibitor, was used, as described in a previous report [26]. In the present study, TSA was injected into the peritoneal cavity of mice 3 days after hydrodynamic injection of pCMV-Huγ, when the transgene expression had dramatically decreased. The administration of TSA had no significant effects on the levels of human IFN-γ (Figure 4). These results exclude the possibility that histone deacetylation is involved in the silencing of the transgene expression of plasmids.

### 3.5. Effect of Partial Hepatectomy on the Transgene Expression of pCpG-Huγ

**Figure 5:**
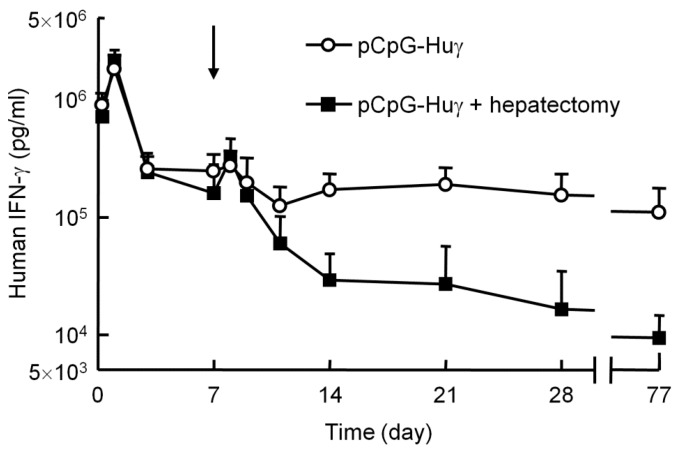
Effect of partial hepatectomy on the serum concentration of human IFN-γ after injection of pCpG-Huγ. pCpG-Huγ was hydrodynamically injected into mice at a dose of 0.1 μg/mouse. On day 7, a group of mice received a partial hepatectomy (indicated with an arrow). The concentrations of human IFN-γ in the serum were measured at the indicated time points. The results are expressed as the mean + SD of four mice.

Partial hepatectomy induces the proliferation of hepatocytes, so that the size of the organ returns to normal within 21 days after the surgery [27]. This procedure has been used to discriminate between integrated and episomal DNA, because the plasmid integrated into the genome is replicated with the proliferation of hepatocytes. Therefore, this technique was used to see whether the pCpG vectors, the persistent expressing plasmid DNA, are integrated into the genome after hydrodynamic injection. About 37% of the liver was removed 7 days after the hydrodynamic injection of pCpG-Huγ. Figure 5 shows the concentration of human IFN-γ in the serum of mice with or without partial hepatectomy. The concentration in the hepatectomy group transiently increased soon after the partial hepatectomy, then decreased with time to a level much lower than that in the control group. The transgene expression was about 10% to 20% of that in the control mice at a steady-state 2 weeks after the partial hepatectomy. These results indicate that pCpG vectors remain in the episomal form, even though they are effective in producing transgenes for a long period of time.

## 4. Discussion

The cytosines in CpG dinucleotides in mammalian DNA are mostly methylated by DNA methyltransferases. Because the methylation generally reduces the efficiency of gene expression, CpG depletion from plasmid vectors has been used to increase the duration of transgene expression [12]. We found in the present study that some CpGs in the promoter region of pCMV-Luc were methylated on and after day 14 post-injection. However, a great reduction in transgene expression of pCMV-Luc after hydrodynamic injection occurs by 3 days [13], when almost no CpG sequences were methylated (Figure 1), strongly suggesting that methylation is not the reason for the reduced transgene expression. Chen *et al.* also proved that CpG methylation status did not change after episomal DNA delivery into mouse liver [28]. On the other hand, we observed that the CpGs in the cDNA are less methylated than those in the promoter region, indicating that the methylation rate depends on the position of the CpGs.

Several research groups, including our own, have reported that the level of transgene expression from plasmid DNA can be significantly upregulated by some stimuli, including, but not limited to, large-volume injection of isotonic solutions, endotoxin, radiation and ischemia/reperfusion. The experiments using pre-methylated plasmid DNA showed that methylated plasmid DNA is much less sensitive to the reactivation stimulus of a large-volume injection (Figure 2). The levels of reactivated transgene expression from methylated pCMV-Luc and pCpG-Luc were lower than those from their unmethylated counterparts with no additional injections. Therefore, a significant level of reactivation of plasmid DNA by a large-volume injection of saline on day 3 (Figure 2) as well as at later time points [2] strongly suggests that pCMV-Luc and other plasmid DNA delivered by hydrodynamic injection are less methylated in mouse liver. An *in vitro* study reported that the intragenic CpG content influenced de novo transcriptional activity, but this regulation was not related to the level of CpG methylation [29]. Moreover, the administration of 5-AZA, an inhibitor of DNA methylation, hardly affected the level of transgene expression from pCMV-Huγ compared with that in the vehicle-treated group (Figure 3). Based on the results obtained so far, the major reason for the reduced transgene expression of CpG-replete plasmid DNA is not CpG methylation of the plasmid DNA. This is consistent with the conclusion made by Chen *et al.*, that transcriptional activity of the transgene expression cassette was not influenced by the CpG methylation status of the plasmid bacterial backbone [28].

Some reports have raised the possibility that histone deacetylation is involved in the reduced transgene expression after gene transfer [20,29,30]. It has been reported that acetylated H3 and H4 histones in mouse liver increased after a single intraperitoneal injection of TSA at a dose of 10 mg/kg [26], and maximal gene activation occurred at approximately 2–4 h post-injection, with a return to base-line by 16 h [26,31]. However, the transgene expression from pCMV-Huγ was unaffected by administration of the same dose of TSA (Figure 4). This is consistent with the conclusion of another study, in which the relationship of histone modification and transgene expression silencing was investigated [5]. Therefore, we believe that the reduced transgene expression from hydrodynamically injected plasmid DNA was not directly caused by the deacetylation of histones.

Another possibility to explain the difference in the duration of transgene expression from CpG-replete and CpG-reduced plasmids involves the integration of plasmids into the genome. Integration of plasmid DNA into host genomic DNA has been challenged using transposon systems [10], because it is considered to increase the duration of transgene expression through integration into the host genome. It has long been known that the level of transgene expression is not significantly reduced by partial hepatectomy when the DNA delivered is integrated into genomic DNA, because hepatectomy-induced cell division increases the number of cells that have the integrated DNA [32,33]. However, the results of the present study showed that the long-expressing plasmid pCpG-Huγ is hardly integrated into genomic DNA (Figure 5).

The silencing of transgene expression from plasmid DNA should be due to complex intracellular events in which multiple factors are involved. Empirical studies have clearly demonstrated that the type of promoter, the bacterial backbone [34] and other epigenetic modifications except DNA methylation and histone deacetylation, such as histone methylation [35,36], at least partly explain the silencing of transgene expression from plasmid DNA.

## 5. Conclusions

We have demonstrated that the reduction in the transgene expression is not due to CpG methylation or histone deacetylation, and that genome integration is not a reason for prolonged transgene expression from CpG-reduced plasmids. These results indicate that the CpG-reduced plasmids are effective vectors that achieve prolonged transgene expression without integration into the host genome, although the CpG sequences in plasmids are less likely to be methylated *in vivo*.
